# *N*-(1*H*-Indol-3-yl­methyl­idene)-4-methyl­piperazin-1-amine

**DOI:** 10.1107/S1600536813028523

**Published:** 2013-10-26

**Authors:** Channappa N. Kavitha, Jerry P. Jasinski, Brian J. Anderson, H. S. Yathirajan, Manpreet Kaur

**Affiliations:** a Department of Studies in Chemistry, University of Mysore, Manasagangotri, Mysore 570 006, India; b Department of Chemistry, Keene State College, 229 Main Street, Keene, NH 03435-2001, USA

## Abstract

In the title compound, C_14_H_18_N_4_, the piperazine ring is in a slightly distorted chair conformation. The indole ring system is twisted from the piperazine ring, making a dihedral angle of 7.27 (11)°. In the crystal, N—H⋯N hydrogen bonds link mol­ecules into chains along [10-1].

## Related literature

For a review of the current pharmacological and toxicological information for piperazine, see: Elliott (2011 ▶). For the biological activity of Schiff base ligands, see: Kharb *et al.* (2012 ▶); Savaliya *et al.* (2010 ▶); Xu *et al.* (2012 ▶). For related structures, see: Guo (2007 ▶); Ming-Lin *et al.* (2007 ▶); Xu *et al.* (2009 ▶); Zhou *et al.* (2011 ▶). For puckering parameters, see Cremer & Pople (1975 ▶). For standard bond lengths, see: Allen *et al.* (1987 ▶).
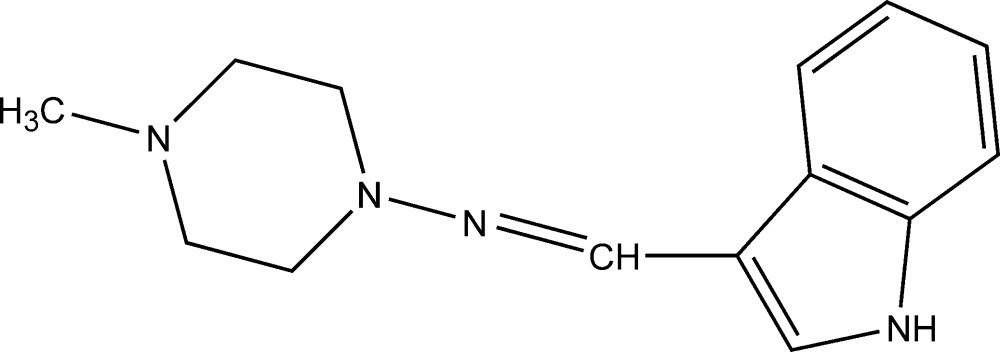


## Experimental

### 

#### Crystal data

C_14_H_18_N_4_
*M* *_r_* = 242.32
Monoclinic, 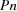

*a* = 7.5630 (5) Å
*b* = 6.5593 (4) Å
*c* = 13.2319 (9) Å
β = 100.072 (6)°
*V* = 646.29 (7) Å^3^
*Z* = 2
Mo *K*α radiation
μ = 0.08 mm^−1^
*T* = 173 K
0.48 × 0.33 × 0.18 mm

#### Data collection

Agilent Gemini Eos diffractometer
Absorption correction: multi-scan *CrysAlis PRO* and *CrysAlis RED*, Agilent (2012 ▶). *T*_min_ = 0.868, *T*_max_ = 1.000
7074 measured reflections
3857 independent reflections
3214 reflections with *I* > 2σ(*I*)
*R*_int_ = 0.050

#### Refinement

*R*[*F* ^2^ > 2σ(*F* ^2^)] = 0.058
*wR*(*F* ^2^) = 0.163
*S* = 1.06
3857 reflections
165 parameters
2 restraints
H-atom parameters constrained
Δρ_max_ = 0.35 e Å^−3^
Δρ_min_ = −0.31 e Å^−3^

### 

Data collection: *CrysAlis PRO* (Agilent, 2012 ▶); cell refinement: *CrysAlis PRO*; data reduction: *CrysAlis RED* (Agilent, 2012 ▶); program(s) used to solve structure: *SUPERFLIP* (Palatinus & Chapuis, 2007 ▶); program(s) used to refine structure: *SHELXL2012* (Sheldrick, 2008 ▶); molecular graphics: *OLEX2* (Dolomanov *et al.*, 2009 ▶); software used to prepare material for publication: *OLEX2*.

## Supplementary Material

Crystal structure: contains datablock(s) I. DOI: 10.1107/S1600536813028523/hg5354sup1.cif

Structure factors: contains datablock(s) I. DOI: 10.1107/S1600536813028523/hg5354Isup2.hkl

Supplementary material file. DOI: 10.1107/S1600536813028523/hg5354Isup3.cml

Additional supplementary materials:  crystallographic information; 3D view; checkCIF report

## Figures and Tables

**Table 1:** Hydrogen-bond geometry (Å, °)

*D*—H⋯*A*	*D*—H	H⋯*A*	*D*⋯*A*	*D*—H⋯*A*
N4—H4⋯N1^i^	0.88	2.29	2.947 (3)	131

Symmetry code: (i) 
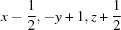
.

**Fig. 1.**
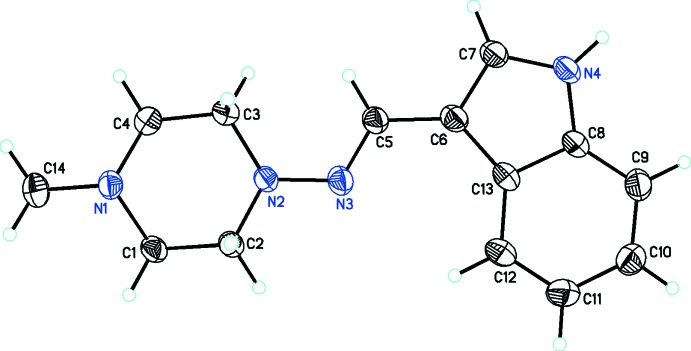
ORTEP drawing of (I) (C14H18N4) showing the labeling scheme with 50% probability displacement ellipsoids.

**Fig. 2.**
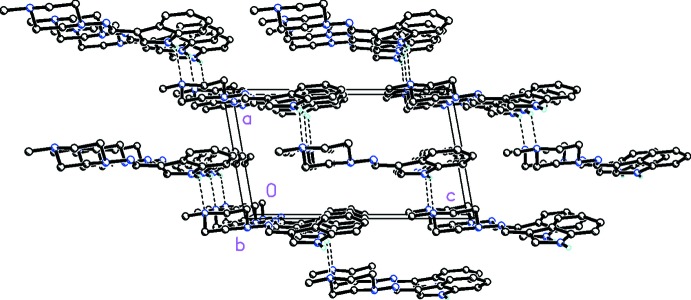
Molecular packing for (I) viewed along the a axis. Dashed lines indicate N—H···N intermolecular hydrogen bonds linking the molecules into chains along [1 0 -1]. H atoms not involved in hydrogen bonding have been removed for clarity.

## supplementary crystallographic information

### 1. Comment

The Schiff base ligands derived from 1-amino-4-methylpiperazine have attracted the interest due to diverse biological applications found with piperazine moiety. Schiff base piperazine derivatives were found to be designed for the study of their antimicrobial activity (Savaliya *et al.*, 2010) and antibacterial activity (Xu *et al.*, 2012). A valuable insight into recent advances on antimicrobial activity of piperazine derivatives is reported (Kharb *et al.*, 2012). A review on the current pharmacological and toxicological information for piperazine derivatives is described (Elliott, 2011). The crystal structures of some related compounds, viz., 2-[(4-methyl piperazin-1-yl)iminomethyl]phenol (Guo, 2007), 1,4-bis{3-[4-(dimethy lamino)benzylideneamino] propyl}piperazine (Xu *et al.*, 2009), 2-methoxy-4-[(4-methylpiperazin-1-yl)-iminomethyl]phenol (Zhou *et al.*, 2011) and 2,4-dibromo-6-[(4-methylpiperazin-1-yl) iminomethyl]phenol (Ming-Lin *et al.*, 2007) have been reported. In view of the above importance of N-piperazinyl Schiff bases, the title compound, (I), C_14_H_18_N_4_, has been synthesized and the crystal structure is reported.

The title compound, (I), crystallizes with one independent molecule in the asymmetric unit (Fig .1). In the molecule, the piperazine ring is in a slightly disordered chair conformation (puckering parameters Q, θ, and φ = 0.568 (3)Å, 175.2 (3)° and 225 (3)°; Cremer & Pople, 1975). The indole ring is twisted from the piperazine ring with a N2/N3/C5/C6 torsion angle of -172.3 (2)°. Bond lengths are in normal ranges (Allen *et al.*, 1987). N—H···N intermolecular hydrogen bonds (Table 1) are observed which link the molecules into chains along [1 0 -1] and influence crystal packing (Fig. 2).

### 2. Experimental

To a solution of indole-3-carboxaldehyde (0.75 g, 0.005 mol) in a mixture of 5 ml of methanol and 5 ml of dichloromethane, an equimolar amount of (1-amino-4-methyl)piperazine (0.57 g, 0.005 mol) is added dropwise with constant stirring. The mixture was refluxed for eight hours to obtain a solution. The solution was evaporated to a small volume at room temperature and allowed to stand. The crystals were formed in one day (m.p.: 459-463 K) and were used as such for x-ray diffraction studies.

### 3. Refinement

All of the H atoms were placed in their calculated positions and then refined using the riding model with Atom—H lengths of 0.95Å (CH), 0.99Å (CH_2_), 0.98Å (CH_3_) or 0.88Å (NH). Isotropic displacement parameters for these atoms were set to 1.2 (CH, CH_2_, NH) or 1.5 (CH_3_) times *U*_eq_ of the parent atom. Idealised Me refined as rotating groups.

### Crystal data

**Table:**

C_14_H_18_N_4_	*F*(000) = 260
*M**_r_* = 242.32	*D*_x_ = 1.245 Mg m^−^^3^
Monoclinic, *P**n*	Mo *K*α radiation, λ = 0.71073 Å
*a* = 7.5630 (5) Å	Cell parameters from 2079 reflections
*b* = 6.5593 (4) Å	θ = 3.4–33.0°
*c* = 13.2319 (9) Å	µ = 0.08 mm^−^^1^
β = 100.072 (6)°	*T* = 173 K
*V* = 646.29 (7) Å^3^	Irregular, yellow
*Z* = 2	0.48 × 0.33 × 0.18 mm

### Data collection

**Table:**

Agilent Gemini Eos diffractometer	3857 independent reflections
Radiation source: Enhance (Mo) X-ray Source	3214 reflections with *I* > 2σ(*I*)
Detector resolution: 16.0416 pixels mm^-1^	*R*_int_ = 0.050
ω scans	θ_max_ = 33.1°, θ_min_ = 3.4°
Absorption correction: multi-scan *CrysAlis PRO* and *CrysAlis RED*, Agilent (2012).	*h* = −9→11
*T*_min_ = 0.868, *T*_max_ = 1.000	*k* = −6→9
7074 measured reflections	*l* = −19→19

### Refinement

**Table:**

Refinement on *F*^2^	Hydrogen site location: inferred from neighbouring sites
Least-squares matrix: full	H-atom parameters constrained
*R*[*F*^2^ > 2σ(*F*^2^)] = 0.058	*w* = 1/[σ^2^(*F*_o_^2^) + (0.0862*P*)^2^] where *P* = (*F*_o_^2^ + 2*F*_c_^2^)/3
*wR*(*F*^2^) = 0.163	(Δ/σ)_max_ < 0.001
*S* = 1.06	Δρ_max_ = 0.35 e Å^−^^3^
3857 reflections	Δρ_min_ = −0.31 e Å^−^^3^
165 parameters	Extinction correction: *SHELXL2012* (Sheldrick, 2008), Fc^*^=kFc[1+0.001xFc^2^λ^3^/sin(2θ)]^-1/4^
2 restraints	Extinction coefficient: 0.027 (9)
Primary atom site location: iterative	

### Special details

**Table:**

Geometry. All esds (except the esd in the dihedral angle between two l.s. planes) are estimated using the full covariance matrix. The cell esds are taken into account individually in the estimation of esds in distances, angles and torsion angles; correlations between esds in cell parameters are only used when they are defined by crystal symmetry. An approximate (isotropic) treatment of cell esds is used for estimating esds involving l.s. planes.

### Fractional atomic coordinates and isotropic or equivalent isotropic displacement parameters (Å^2^)

**Table:**

	*x*	*y*	*z*	*U*_iso_*/*U*_eq_	
N1	0.5403 (3)	1.0302 (3)	0.32552 (17)	0.0256 (4)	
N2	0.4353 (3)	0.8708 (3)	0.50758 (17)	0.0256 (5)	
N3	0.4539 (3)	0.7746 (4)	0.60330 (19)	0.0283 (5)	
N4	0.3483 (3)	0.2381 (3)	0.8128 (2)	0.0289 (5)	
H4	0.3168	0.1197	0.8355	0.035*	
C1	0.5358 (4)	1.1586 (4)	0.4158 (2)	0.0262 (5)	
H1A	0.4167	1.2248	0.4097	0.031*	
H1B	0.6279	1.2668	0.4196	0.031*	
C2	0.5711 (3)	1.0305 (4)	0.5122 (2)	0.0252 (5)	
H2A	0.6918	0.9678	0.5194	0.030*	
H2B	0.5686	1.1181	0.5728	0.030*	
C3	0.4253 (4)	0.7427 (4)	0.4164 (2)	0.0270 (5)	
H3A	0.3232	0.6470	0.4125	0.032*	
H3B	0.5369	0.6615	0.4214	0.032*	
C4	0.4012 (3)	0.8721 (4)	0.3205 (2)	0.0287 (5)	
H4A	0.4052	0.7834	0.2604	0.034*	
H4B	0.2816	0.9379	0.3108	0.034*	
C5	0.4143 (4)	0.5839 (4)	0.6077 (2)	0.0271 (5)	
H5	0.3872	0.5075	0.5459	0.032*	
C6	0.4101 (4)	0.4842 (4)	0.7047 (2)	0.0268 (5)	
C7	0.3487 (4)	0.2858 (4)	0.7119 (2)	0.0276 (5)	
H7	0.3125	0.1967	0.6555	0.033*	
C8	0.4050 (3)	0.4047 (4)	0.8729 (2)	0.0256 (5)	
C9	0.4229 (4)	0.4299 (5)	0.9786 (2)	0.0329 (6)	
H9	0.3953	0.3224	1.0214	0.039*	
C10	0.4827 (4)	0.6176 (5)	1.0192 (2)	0.0368 (6)	
H10	0.4946	0.6398	1.0910	0.044*	
C11	0.5260 (4)	0.7757 (5)	0.9559 (2)	0.0358 (7)	
H11	0.5679	0.9023	0.9858	0.043*	
C12	0.5083 (4)	0.7494 (4)	0.8510 (3)	0.0302 (6)	
H12	0.5377	0.8569	0.8088	0.036*	
C13	0.4467 (3)	0.5622 (4)	0.8078 (2)	0.0247 (5)	
C14	0.5122 (4)	1.1517 (5)	0.2313 (2)	0.0363 (7)	
H14A	0.3941	1.2173	0.2226	0.054*	
H14B	0.5179	1.0628	0.1724	0.054*	
H14C	0.6058	1.2563	0.2359	0.054*	

### Atomic displacement parameters (Å^2^)

**Table:**

	*U*^11^	*U*^22^	*U*^33^	*U*^12^	*U*^13^	*U*^23^
N1	0.0311 (10)	0.0236 (10)	0.0232 (10)	0.0024 (9)	0.0083 (8)	0.0033 (8)
N2	0.0341 (11)	0.0196 (9)	0.0250 (11)	−0.0007 (9)	0.0107 (8)	0.0011 (8)
N3	0.0346 (11)	0.0256 (11)	0.0272 (11)	0.0005 (9)	0.0121 (9)	0.0022 (9)
N4	0.0360 (12)	0.0201 (10)	0.0321 (12)	−0.0038 (9)	0.0098 (9)	0.0035 (9)
C1	0.0328 (12)	0.0182 (10)	0.0290 (13)	0.0009 (10)	0.0093 (10)	0.0015 (10)
C2	0.0313 (12)	0.0203 (11)	0.0256 (13)	−0.0021 (9)	0.0098 (9)	0.0002 (9)
C3	0.0328 (13)	0.0219 (11)	0.0269 (13)	−0.0018 (10)	0.0070 (10)	−0.0010 (10)
C4	0.0314 (12)	0.0290 (13)	0.0257 (13)	−0.0003 (10)	0.0052 (10)	−0.0005 (10)
C5	0.0332 (12)	0.0232 (12)	0.0271 (13)	−0.0017 (10)	0.0116 (10)	−0.0002 (10)
C6	0.0300 (12)	0.0224 (11)	0.0301 (13)	−0.0019 (9)	0.0112 (9)	0.0001 (10)
C7	0.0320 (12)	0.0223 (11)	0.0303 (14)	−0.0016 (10)	0.0101 (10)	0.0002 (10)
C8	0.0258 (11)	0.0207 (11)	0.0303 (13)	−0.0013 (10)	0.0048 (9)	0.0037 (10)
C9	0.0337 (13)	0.0340 (14)	0.0299 (14)	−0.0037 (12)	0.0026 (10)	0.0072 (12)
C10	0.0375 (15)	0.0410 (16)	0.0299 (15)	−0.0053 (13)	0.0002 (11)	0.0015 (12)
C11	0.0359 (15)	0.0325 (14)	0.0380 (17)	−0.0076 (12)	0.0040 (12)	−0.0043 (12)
C12	0.0268 (11)	0.0255 (12)	0.0388 (15)	−0.0048 (10)	0.0072 (10)	0.0011 (11)
C13	0.0217 (10)	0.0215 (11)	0.0318 (13)	0.0004 (9)	0.0073 (9)	0.0048 (10)
C14	0.0454 (16)	0.0343 (15)	0.0293 (14)	−0.0004 (13)	0.0069 (12)	0.0098 (12)

### Geometric parameters (Å, º)

**Table:**

N1—C1	1.467 (3)	C4—H4B	0.9900
N1—C4	1.470 (3)	C5—H5	0.9500
N1—C14	1.463 (4)	C5—C6	1.446 (4)
N2—N3	1.400 (3)	C6—C7	1.390 (4)
N2—C2	1.461 (3)	C6—C13	1.438 (4)
N2—C3	1.462 (3)	C7—H7	0.9500
N3—C5	1.289 (4)	C8—C9	1.392 (4)
N4—H4	0.8800	C8—C13	1.416 (4)
N4—C7	1.371 (4)	C9—H9	0.9500
N4—C8	1.375 (4)	C9—C10	1.387 (4)
C1—H1A	0.9900	C10—H10	0.9500
C1—H1B	0.9900	C10—C11	1.407 (5)
C1—C2	1.511 (4)	C11—H11	0.9500
C2—H2A	0.9900	C11—C12	1.382 (4)
C2—H2B	0.9900	C12—H12	0.9500
C3—H3A	0.9900	C12—C13	1.399 (4)
C3—H3B	0.9900	C14—H14A	0.9800
C3—C4	1.510 (4)	C14—H14B	0.9800
C4—H4A	0.9900	C14—H14C	0.9800
			
C1—N1—C4	108.8 (2)	N3—C5—H5	119.3
C14—N1—C1	111.1 (2)	N3—C5—C6	121.3 (3)
C14—N1—C4	110.5 (2)	C6—C5—H5	119.3
N3—N2—C2	109.1 (2)	C7—C6—C5	122.9 (3)
N3—N2—C3	118.0 (2)	C7—C6—C13	106.2 (2)
C2—N2—C3	112.4 (2)	C13—C6—C5	130.6 (2)
C5—N3—N2	119.4 (2)	N4—C7—C6	109.8 (2)
C7—N4—H4	125.4	N4—C7—H7	125.1
C7—N4—C8	109.1 (2)	C6—C7—H7	125.1
C8—N4—H4	125.4	N4—C8—C9	129.9 (3)
N1—C1—H1A	109.7	N4—C8—C13	108.0 (2)
N1—C1—H1B	109.7	C9—C8—C13	122.1 (3)
N1—C1—C2	110.0 (2)	C8—C9—H9	121.2
H1A—C1—H1B	108.2	C10—C9—C8	117.6 (3)
C2—C1—H1A	109.7	C10—C9—H9	121.2
C2—C1—H1B	109.7	C9—C10—H10	119.4
N2—C2—C1	110.1 (2)	C9—C10—C11	121.2 (3)
N2—C2—H2A	109.6	C11—C10—H10	119.4
N2—C2—H2B	109.6	C10—C11—H11	119.5
C1—C2—H2A	109.6	C12—C11—C10	120.9 (3)
C1—C2—H2B	109.6	C12—C11—H11	119.5
H2A—C2—H2B	108.2	C11—C12—H12	120.4
N2—C3—H3A	109.5	C11—C12—C13	119.1 (3)
N2—C3—H3B	109.5	C13—C12—H12	120.4
N2—C3—C4	110.6 (2)	C8—C13—C6	106.9 (2)
H3A—C3—H3B	108.1	C12—C13—C6	134.0 (3)
C4—C3—H3A	109.5	C12—C13—C8	119.1 (3)
C4—C3—H3B	109.5	N1—C14—H14A	109.5
N1—C4—C3	112.2 (2)	N1—C14—H14B	109.5
N1—C4—H4A	109.2	N1—C14—H14C	109.5
N1—C4—H4B	109.2	H14A—C14—H14B	109.5
C3—C4—H4A	109.2	H14A—C14—H14C	109.5
C3—C4—H4B	109.2	H14B—C14—H14C	109.5
H4A—C4—H4B	107.9		
			
N1—C1—C2—N2	−59.6 (3)	C5—C6—C13—C12	−5.0 (5)
N2—N3—C5—C6	−172.3 (2)	C7—N4—C8—C9	178.7 (3)
N2—C3—C4—N1	54.1 (3)	C7—N4—C8—C13	−1.3 (3)
N3—N2—C2—C1	−171.0 (2)	C7—C6—C13—C8	0.0 (3)
N3—N2—C3—C4	178.7 (2)	C7—C6—C13—C12	−179.1 (3)
N3—C5—C6—C7	172.6 (3)	C8—N4—C7—C6	1.4 (3)
N3—C5—C6—C13	−0.8 (4)	C8—C9—C10—C11	−0.9 (4)
N4—C8—C9—C10	−179.5 (3)	C9—C8—C13—C6	−179.2 (2)
N4—C8—C13—C6	0.8 (3)	C9—C8—C13—C12	0.1 (4)
N4—C8—C13—C12	−179.9 (2)	C9—C10—C11—C12	0.7 (5)
C1—N1—C4—C3	−57.8 (3)	C10—C11—C12—C13	−0.1 (4)
C2—N2—N3—C5	−148.2 (2)	C11—C12—C13—C6	178.8 (3)
C2—N2—C3—C4	−53.0 (3)	C11—C12—C13—C8	−0.3 (4)
C3—N2—N3—C5	−18.4 (3)	C13—C6—C7—N4	−0.9 (3)
C3—N2—C2—C1	56.1 (3)	C13—C8—C9—C10	0.5 (4)
C4—N1—C1—C2	60.0 (3)	C14—N1—C1—C2	−178.1 (2)
C5—C6—C7—N4	−175.6 (2)	C14—N1—C4—C3	179.9 (2)
C5—C6—C13—C8	174.2 (3)		

### Hydrogen-bond geometry (Å, º)

**Table:**

*D*—H···*A*	*D*—H	H···*A*	*D*···*A*	*D*—H···*A*
N4—H4···N1^i^	0.88	2.29	2.947 (3)	131

Symmetry code: (i) *x*−1/2, −*y*+1, *z*+1/2.
